# Benefits of Multi-Constellation/Multi-Frequency GNSS in a Tightly Coupled GNSS/IMU/Odometry Integration Algorithm †

**DOI:** 10.3390/s18093052

**Published:** 2018-09-12

**Authors:** Björn Reuper, Matthias Becker, Stefan Leinen

**Affiliations:** Department of Physical and Satellite Geodesy, Technische Universität Darmstadt, Franziska-Braun-Straße 7, 64287 Darmstadt, Germany; becker@psg.tu-darmstadt.de (M.B.); leinen@psg.tu-darmstadt.de (S.L.)

**Keywords:** global navigation satellite systems, GNSS, inertial measurement unit, IMU, odometry, tightly coupled, multi-frequency, multi-constellation

## Abstract

Localization algorithms based on global navigation satellite systems (GNSS) play an important role in automotive positioning. Due to the advent of autonomously driving cars, their importance is expected to grow even further in the next years. Simultaneously, the performance requirements for these localization algorithms will increase because they are no longer used exclusively for navigation, but also for control of the vehicle’s movement. These requirements cannot be met with GNSS alone. Instead, algorithms for sensor data fusion are needed. While the combination of GNSS receivers with inertial measurements units (IMUs) is a common approach, it is traditionally executed in a single-frequency/single-constellation architecture, usually with the Global Positioning System’s (GPS) L1 C/A signal. With the advent of new GNSS constellations and civil signals on multiple frequencies, GNSS/IMU integration algorithm performance can be improved by utilizing these new data sources. To achieve this, we upgraded a tightly coupled GNSS/IMU integration algorithm to process measurements from GPS (L1 C/A, L2C, L5) and Galileo (E1, E5a, E5b). After investigating various combination strategies, we chose to preferably work with ionosphere-free combinations of L5-L1 C/A and E5a-E1 pseudo-ranges. L2C-L1 C/A and E5b-E1 combinations as well as single-frequency pseudo-ranges on L1 and E1 serve as backup when no L5/E5a measurements are available. To be able to process these six types of pseudo-range observations simultaneously, the differential code biases (DCBs) of the employed receiver need to be calibrated. Time-differenced carrier-phase measurements on L1 and E1 provide the algorithm with pseudo-range-rate observations. To provide additional aiding, information about the vehicle’s velocity obtained by an odometry model fed with angular velocities from all four wheels as well as the steering wheel angle is incorporated into the algorithm. To evaluate the performance improvement provided by these new data sources, two sets of measurement data are collected and the resulting navigation solutions are compared to a higher-grade reference system, consisting of a geodetic GNSS receiver for real-time kinematic positioning (RTK) and a navigation grade IMU. The multi-frequency/multi-constellation algorithm with odometry aiding achieves a 3-D root mean square (RMS) position error of 3.6 m/2.1 m in these data sets, compared to 5.2 m/2.9 m for the single-frequency GPS algorithm without odometry aiding. Odometry is most beneficial to positioning accuracy when GNSS measurement quality is poor. This is demonstrated in data set 1, resulting in a reduction of the horizontal position error’s 95% quantile from 6.2 m without odometry aiding to 4.2 m with odometry aiding.

## 1. Introduction

Localization algorithms for automotive applications are predominantly based on *global navigation satellite systems* (GNSS). Since road vehicles frequently travel in surroundings with poor satellite visibility (urban canyons, tunnels, etc.), additional sensors providing information about the vehicle’s position and/or movement are commonly used. The integration of GNSS and *inertial measurement units* (IMUs) in a fusion filter is the most prominent approach, often in combination with even more sensors (e.g., odometry, cameras, etc.). In tightly coupled GNSS/IMU integration algorithms, the sensor data fusion is performed in the range domain. The fusion filter inputs pseudo-range and pseudo-range-rate measurements from the GNSS receiver and utilizes them to correct position, velocity and attitude estimates derived from IMU measurements. Additional outputs of the fusion filter are IMU errors together with the GNSS receiver clock bias and drift [1]. Traditionally, the only GNSS signal available to civil users was the *Global Positioning System’s* (GPS) L1 C/A signal. With the advent of new GNSS constellations and civil signals on multiple frequencies, GNSS/IMU integration algorithm performance can be improved by utilizing these new data sources. This introduces new effects into the localization algorithm that need to be addressed. Some of these effects are the time offset between the different GNSS time scales [2], the difference between the receiver hardware delays affecting the signals from different GNSS (*inter-system bias*, ISB [3]) and *differential code biases* (DCBs [4]). To include further aiding to the integrated navigation solution, odometry models are frequently employed. In common integration schemes, the models are based on a single wheel speed sensor measuring the angular velocity of an undriven, unsteered axle and/or non-holonomic constraints modeling the vehicle’s dynamics [5,6,7,8]. These schemes neglect longitudinal and lateral wheel slip and cannot aid the localization algorithm reliably whenever the vehicle is accelerating, decelerating or cornering.

In this paper, we present an approach to deal with these challenges in a tightly coupled GNSS/IMU/odometry integration algorithm. The following Section 2 concerns the integration of GNSS measurements into the algorithm. In the beginning, an integration algorithm that works with GPS L1 C/A code pseudo-ranges and time-differenced carrier-phase measurements as the only GNSS data is presented (Section 2.1). The first upgrade step is the inclusion of pseudo-ranges derived from additional civil GPS signals on different frequencies (L2C and L5) in Section 2.2. Afterwards, Galileo signals on E1, E5a and E5b are added to the localization algorithm in Section 2.3. The utilization of pseudo-range observations on multiple carrier frequencies enables the elimination of the ionospheric error. Time-differenced carrier-phase measurements do not exhibit substantial ionospheric errors because these are removed by time-differencing. Consequently, we continue to use carrier-phase observations for GPS on L1 only and add merely the ones on E1 for Galileo. Section 3 describes an extended odometry model capable of estimating longitudinal and lateral velocity of all wheels individually. Since DCBs constitute a major problem for the combination of pseudo-ranges from different frequencies, Section 4 outlines a method to calibrate them and shows the calibration results for one type of GNSS receiver. To evaluate the performance of the upgraded algorithm in comparison to the basic one, the estimation errors of the algorithm versions are presented in Section 5. These estimation errors are obtained from differences to a higher-grade reference system, consisting of a geodetic GNSS receiver for *real-time kinematic positioning* (RTK) and a navigation grade IMU. Finally, we discuss our conclusions in Section 6.

## 2. Utilization of GNSS

This section describes how GNSS measurements are utilized in different development stages of the localization algorithm. Starting from GPS L1 C/A code pseudo-ranges and time-differenced carrier-phase observations only (Section 2.1), multi-frequency GPS measurements are incorporated in Section 2.2 to enhance accuracy by eliminating the ionospheric error. In the concluding Section 2.3, multi-frequency Galileo observations are added to increase satellite availability.

### 2.1. Basic GPS L1 Algorithm

The basic algorithm is a tightly coupled GPS/IMU fusion filter. The central component is a closed-loop error-state *Extended Kalman Filter* (EKF) with 17 states (see Table 1). Errors in attitude, velocity and position are resolved in the local navigation coordinate frame (indicated by the superscript *n*) with the order *east-north-up* (ENU). Errors in gyroscope and accelerometer offset are resolved in the body coordinate frame (indicated by the superscript *b*) with the order *front-left-up* (FLU). The subscripts of the first nine states refer to the two coordinate frames being referenced to each other. For example, $v_{en}^{n}$ is the velocity of the navigation frame *n* with respect to the earth-fixed frame *e*, expressed in navigation frame coordinates. Errors in receiver clock bias δt and drift $\delta \dot{t}$ are multiplied by the speed of light *c* to get units of meters and meters per second, respectively.

The localization algorithm inputs 3-D angular velocities and accelerations from a *microelectromechanical systems* (MEMS) IMU as well as pseudo-ranges ρ and carrier-phase measurements ϕ from the GPS receiver. The carrier-phase measurements are time-differenced to remove the phase ambiguity. This results in pseudo-range-rate observations $\dot{\rho }$ which are input into the EKF for velocity determination. Since these pseudo-range-rates are derived from carrier-phase measurements at two consecutive epochs, they are applied to the navigation solution with a time stamp in the middle between these two epochs.

The localization algorithm operates in two different modes: initialization and normal mode. For initialization of the EKF’s states, different sources of information are used. In addition to the state estimates, *standard deviations* (std.) to model the initialization values’ accuracy are provided as well:

- Roll and pitch angle are estimated from IMU-measured accelerations. Based on offset and noise characteristics of the IMU in use, the standard deviation is set to $3\sigma =5^{\circ }$.
- The yaw angle is estimated from the GPS-derived velocity under the assumption that the vehicle is traveling in a straight line without side slip. Based on velocity estimation quality of the GPS-receiver used here, a standard deviation of $3\sigma =10^{\circ }$ is employed.
- Velocity and receiver clock drift are estimated based on pseudo-range-rate observations $\dot{\rho }$. The pseudo-range-rates’ variance is set to $\sigma_{\dot{\rho }}^{2}=0.1\;m^{2}/s^{2}$.
- Position and receiver clock bias are estimated from pseudo-ranges via a single-epoch navigation solution. The pseudo-ranges’ variance $\sigma_{\rho }^{2}$ is modeled as the sum of two parts: $\sigma_{\rho ,\theta }^{2}$, depending on the satellite’s elevation θ, and $\sigma_{\rho ,C/N_{0}}^{2}$, depending on the signal’s carrier to noise ratio $C/N_{0}$.
- Gyroscope and accelerometer offset are initialized as 0. Their initial variance is based on the nominal values for the bias repeatability given by the IMU’s manufacturer.

Once initialization is completed, the algorithm enters normal operation mode. Whenever a new set of IMU measurements is received, the estimated values of attitude, velocity and position are updated in a strapdown algorithm. Simultaneously, the state vector’s covariance matrix is propagated forward in time in the EKF. When new measurements from the GPS receiver are available, they get processed in two steps:

1. Preprocessing: Based on the a-posteriori values of receiver clock bias and drift from the last time step, the a-priori values of these quantities are propagated. Corrections for satellite and receiver clock errors as well as ionospheric and tropospheric refraction are applied to pseudo-ranges and pseudo-range-rates. The measurement noise covariance for pseudo-range and pseudo-range-rate measurements is calculated. All measurements are assumed to be uncorrelated with each other; the variances for pseudo-ranges and pseudo-range-rates are the same as in initialization mode. Lastly, positions and velocities of the received satellites are computed.
2. Measurement update: The innovation δz is formed as difference between the corrected pseudo-range and pseudo-range-rate measurements and their predicted counterparts. These predictions are based on the a-priori estimates of attitude, velocity and position. Plausibility checks for pseudo-ranges and pseudo-range-rates are employed to detect outliers. Finally, δz and the associated measurement noise covariance are used to determine corrections for the state vector’s a-priori estimates. Simultaneously, the state vector’s covariance matrix is updated to reflect the newly incorporated information.

### 2.2. Multi-Frequency GPS Algorithm

The overall pseudo-range error, often called *user equivalent range error* (UERE), is usually decomposed into two error types which are considered statistically independent: The *signal in space ranging error* (SISRE) and the *user equipment error* (UEE) [9].

(1) $$\mathrm{UERE}=\sqrt{{\mathrm{SISRE}}^{2}+{\mathrm{UEE}}^{2}}$$

Modern (circa 2016 and later) dual-frequency receivers provide a typical *root mean square* (RMS) ionosphere error of 0.4 m, resulting in a UEE of 0.5 m and a UERE of 0.7 m (assuming no significant multipath error). In comparison, the typical RMS ionosphere error of modern single-frequency receivers is 5.0 m. As all other errors are significantly smaller, this results in both a UEE and a UERE of 5.0 m [9]. Hence, multi-frequency signals accessible to civil users provide the potential to drastically increase measurement accuracy. Out of a total of 31 active GPS satellites, the GPS L2C signal today is available from 19 satellites (7 block IIR-M and 12 block IIF), while the GPS L5 signal is available from the 12 block IIF satellites [10]. With the upcoming launches of block IIIA GPS satellites, both numbers will increase. For Galileo (see Section 2.3), availability of civil multi-frequency signals is not an issue as all satellites provide civil signals on E1, E5a and E5b.

To input pseudo-range measurements on additional frequencies into the localization algorithm, the following alternatives were considered:

1. Treat the new measurements exactly as the old ones, i.e. retain the assumption that all measurements are uncorrelated and assign similar variances to the new pseudo-range measurements as to the old ones.
2. Treat measurements stemming from the same satellite as a batch, i.e. assign similar variances to the new pseudo-range measurements as to the old ones but assume that pseudo-range measurements on different frequencies, but from the same satellite, are highly correlated. Pseudo-ranges from different satellites are still considered to be uncorrelated.
3. Introduce additional variables into the EKF’s state vector to estimate the ionospheric refraction. The simplest version of this alternative adds a single additional state representing the zenith ionospheric delay on a reference carrier frequency. The ionospheric refraction’s dependency on carrier frequency and elevation is represented in the pseudo-range measurement model. More complex versions add one state for each satellite in view, resulting in a variable-length state vector.
4. Work with ionosphere-free linear combinations of pseudo-ranges.

Option (1) does not model the ionospheric refraction’s dependency on the carrier frequency and is therefore clearly suboptimal. Option (2) does take this dependency into account. However, this is only done implicitly via the off-diagonal entries in the measurement noise covariance matrix. The result is an increased computational load as some of the measurements are now correlated to each other. Also, parametrizing the measurement noise covariance matrix becomes more difficult. While option (3) provides a high degree of flexibility, it also increases the algorithm’s complexity significantly due to the additional variables in the state vector. Ionosphere-free linear combinations (option 4) offer the advantage of being able to keep many of the existing algorithm’s features: Measurements from different satellites can still be considered uncorrelated and the EKF’s state vector does not need to be changed. Also, the properties of ionosphere-free combinations are well known because they have been investigated since the launch of GPS, they are the preferred mode for ionospheric corrections according to the GPS interface specifications and the *civil navigation messages* (CNAV) on L2C and L5 provide terms to correct for the effects of satellite-specific DCBs in the respective combinations [11,12]. On the other hand, ionosphere-free linear combinations only support dual-frequency measurements and not three or even more measurement frequencies. They also suffer from increased code tracking noise [1]:

(2) $$\sigma_{\rho ,IF}=\frac{\sqrt{f_{\alpha }^{4}\sigma_{\rho ,\alpha }^{2}+f_{\beta }^{4}\sigma_{\rho ,\beta }^{2}}}{\left|f_{\alpha }^{2}+f_{\beta }^{2}\right|}$$

where $\sigma_{\rho ,IF}$, $\sigma_{\rho ,\alpha }$ and $\sigma_{\rho ,\beta }$ are the code tracking error standard deviations for the ionosphere-free combination (IF), the first signal (α) and the second signal (β), and $f_{\alpha }$ and $f_{\beta }$ are the first and second signal’s carrier frequencies. When taking into account the slightly different code tracking error standard deviations due to different transmission powers for each signal, this results in an amplification of the standard deviation by a factor of ca. 3.36 (for L2C-L1 C/A combinations) or ca. 2.59 (for L5-L1 C/A combinations) in comparison to single-frequency L1 C/A measurements [1].

We decided to implement option (4) because it offers the main advantage of multi-frequency measurements (eliminating the ionospheric error almost completely) while only requiring changes to the algorithm’s GPS preprocessing and not to the EKF and its state vector. The lack of support for three or more carrier frequencies is not a significant issue, as the inclusion of more than two frequencies offers diminishing performance improvements when compared to the improvement achieved by integrating a second frequency. As the Kalman Filter possesses an inherent smoothing ability, it attenuates the increased measurement noise’s effect, hence mitigating the second disadvantage of ionosphere-free linear combinations.

According to the GPS interface specifications [11,12], the ionosphere-free linear combinations based on CNAV data for L1 C/A, L2C, L5I and L5Q are:

(3) $$\rho_{IF}=\frac{\left(\rho_{\alpha }-\gamma_{\beta ,\alpha }\rho_{\beta }\right)+c\left({\mathrm{ISC}}_{\alpha }-\gamma_{\beta ,\alpha }{\mathrm{ISC}}_{\beta }\right)}{1-\gamma_{\beta ,\alpha }}-cT_{GD}$$

where α and β stand for either L1 C/A, L2C, L5I or L5Q. $\gamma_{\beta ,\alpha }$ is the squared ratio between the respective carrier frequencies, *c* is the speed of light, ${\mathrm{ISC}}_{i}$ is the *inter-signal correction term* for the channel indicated by the subscript *i* (equivalent to the term “satellite-specific DCB” [4]) and $T_{GD}$ is the timing group delay from the GPS *legacy navigation message* (LNAV). Accessing the ISCs is problematic as the *Receiver Independent Exchange Format* (RINEX) in its current version 3.03 only supports GPS LNAV data [13]. To avoid these restrictions, a CNAV-compatible RINEX-style format developed for the CNAV test campaign in 2013 [14] and the corresponding navigation files provided by the *Crustal Dynamics Data Information System* (CDDIS) [15] are used. For the calculation of satellite positions, the ephemeris provided by LNAV continue to be used in order to preserve compatibility with block IIR and earlier GPS satellites as well as Galileo, whose ephemeris representation is identical to GPS LNAV.

Initially, an algorithm capable of working with L2C-L1 C/A combinations, L5I-L1 C/A combinations, L5Q-L1 C/A combinations and single-frequency L1 C/A measurements simultaneously without considering DCBs was implemented. Based on the available pseudo-range measurements at each epoch, the GPS preprocessing decides what data it forwards to the EKF and computes a matching variance. From most favorable to least favorable, the hierarchy is L5Q-L1 C/A > L5I-L1 C/A > L2C-L1 C/A > L1 C/A. Higher preference was given to L5 compared to L2C because L5 offers a larger frequency difference to L1 and the L5/L1 carrier frequencies match those of Galileo E5a/E1. The variance of all pseudo-range measurements is given by:

(4) $$\sigma_{\rho }^{2}=\sigma_{\rho ,\theta }^{2}+\sigma_{\rho ,C/N_{0}}^{2}-\sigma_{\rho ,iono}^{2}$$

where the first two terms $\sigma_{\rho ,\theta }^{2}$ and $\sigma_{\rho ,C/N_{0}}^{2}$ are the same as they were for the basic GPS L1 algorithm. The variance reduction term $\sigma_{\rho ,iono}^{2}$ describes the magnitude of the accuracy improvement due to the elimination of ionospheric errors. It is 0 for single-frequency measurements and is assigned according to (5) for ionosphere-free combinations.

(5) $$\sigma_{\rho ,iono}^{2}=\left\{\begin{array}{cc} 25\;m^{2}, & \mathrm{for}\;\theta \geq 20^{\circ }\\ \frac{\sin20^{\circ }}{\sin\theta }\;\cdot \;25\;m^{2}, & \mathrm{for}\;\theta <20^{\circ } \end{array}\right.$$

This way, the ionosphere-free linear combinations get assigned a lower measurement variance than single-frequency pseudo-ranges. This contradicts (2), which specifies an increased standard deviation of ionosphere-free linear combinations compared to single-frequency pseudo-ranges. The contradiction is resolved by looking at the Kalman Filter’s assumptions for measurement noise: zero-mean, Gaussian and uncorrelated in time. Noise of this type is fully specified by its covariance matrix. Unfortunately, none of these assumptions is true for pseudo-range measurements. Consequently, the measurement noise’s variance is increased, especially to account for the nonzero mean of pseudo-range errors. The mean error’s absolute value for ionosphere-free combinations is substantially smaller than for single-frequency measurements, because the ionospheric error is the largest contributor to the overall error. That is why the ionosphere-free combinations are modeled with smaller measurement noise variance as far as the Kalman Filter is concerned.

While the measurement noise variance model turned out to be feasible, the idea to work with all possible ionosphere-free linear combinations as well as single-frequency pseudo-ranges without taking the DCBs into account did not. Firstly, the GNSS receiver used (a JAVAD TRIUMPH-LS) does not track the in-phase and quadra-phase component on L5 independently, but uses a combined I + Q tracking. The resulting pseudo-ranges receive the observation code C5X in RINEX 3.03 [13]. No ISCs are provided in the CNAV data for this type of code tracking, so the satellite-specific DCBs cannot be eliminated in the way they could for independent I and Q tracking. In the following results, GPS C5X pseudo-ranges are processed with the ISCs for GPS L5I. Secondly, the receiver-specific DCBs proved to be too large to facilitate a composition of all available ionosphere-free combinations and single-frequency pseudo-ranges without DCB calibration (see Section 4).

Table 2 summarizes the receiver clock biases obtained when working with different types of observations, all stemming from the same three data sets. $c\delta t_{L1\;C/A}$, the receiver clock bias obtained from the EKF outputs when only processing L1 C/A pseudo-ranges, is treated as reference. The entries in the second column are gained by computing the mean and standard deviation of the differences $c\delta t_{L1\;C/A}-c\delta t_{i}$, where the subscript *i* indicates the type of processed code observations and is specified in the first column. The first line in each cell specifies the mean, the second line specifies the standard deviation. The three entries in each line stem from three different sets of measurement data, collected between December 2016 and November 2017. All three data sets contain kinematic data and have durations of approximately 120 min/60 min/30 min while covering distances of approximately 100 km/13 km/8 km.

As the code biases in their non-differential form cannot be separated from the receiver clock bias, the difference in receiver clock bias is an indication for the DCBs. The last column ranks the positioning performance in each data set from best (1) to worst (7), based on the RMS position error. For two of the three data sets, it was not possible to compute a solution with L5-L1 C/A combinations alone, because there were not enough block IIF satellites in view. The first data set contains just barely enough block IIF satellites, but the low number of pseudo-range measurements results in the highest standard deviation for the receiver clock bias difference as well as the worst positioning performance. The L2C-L1 C/A combinations exhibit large differences in receiver clock bias when compared to the single-frequency L1 C/A measurements (up to −7.5 m). Consequently, combining these two types of observations results in degraded positioning performance. The third data set is an exception and offers very good positioning performance when processing both L2C-L1 C/A combinations and single-frequency L1 C/A measurements together. This happens because while recording this data set, only very few older GPS satellites (block IIR and earlier) were received. Most of the visible satellites provided L2C signals, causing the solution to be close to the one in which only L2C-L1 C/A combinations were processed. While the difference in estimated receiver clock bias could not be computed reliably when only processing L5-L1 C/A combinations, the low values of mean and standard deviation as well as the good positioning performance when processing both L5-L1 C/A combinations and single-frequency L1 C/A measurements together indicates that there are no significant DCBs for these two types of observations. The poor positioning performance whenever both L2C-L1 C/A and L5-L1 C/A combinations are processed together suggests that substantial DCBs between these two types of observations exist. Subsequent DCB calibration (see Section 4) substantiates these hypotheses.

### 2.3. Multi-GNSS Algorithm (GPS and Galileo)

Road vehicles frequently travel in surroundings with partially obstructed sky view. These obstructions include buildings, trees, bridges and other vehicles. Consequently, the number of received satellites is smaller than it would be under an unobstructed sky. Received signals may suffer from reduced $C/N_{0}$ and increased multipath due to signal reflections on surrounding surfaces. During periods of limited satellite visibility, enabling the localization algorithm to process signals from additional GNSS constellations mitigates these negative effects by increasing the number of available satellites.

Galileo implements the same ephemeris representation as GPS LNAV. It is also a *code division multiple access* (CDMA) system and shares two common carrier frequencies with GPS: L1/E1 at 1575.42 MHz and L5/E5a at 1176.45 MHz. All Galileo satellites broadcast civil signals on multiple frequencies. These characteristics make Galileo an ideal choice for a second GNSS to work together with GPS in automotive positioning.

To enable Galileo processing, both the preprocessing routine and the EKF were changed slightly. The EKF’s state vector was extended by one entry, representing the Galileo receiver clock bias (see Table 3). Although the same receiver is used for both GNSS, the additional state is necessary to account for the offset between the different GNSS time scales as well as a receiver-specific ISB. Alternatively, 17 states could be maintained and the timing differences between GPS and Galileo could be compensated via the *GPS to Galileo Time Offset* (GGTO) extracted from one of the navigation messages or the *GPS to Galileo time system correction* (GPGA) in the respective RINEX files. This approach did not succeed as the receiver-specific timing bias is too large to be ignored and the parameters in the navigation messages only address the satellite-specific timing bias. During the measurement update, pseudo-ranges and pseudo-range-rates to GPS satellites affect states 16 and 17, while pseudo-ranges and pseudo-range-rates to Galileo satellites affect states 18 and 17. All parameters concerning the EKF’s stochastic model (e.g., $\sigma_{\rho }^{2}$, $\sigma_{\dot{\rho }}^{2}$, system and initial state covariance matrices) are set to the same values for Galileo as they are for GPS. Since the additional state needs to be initialized, the single-epoch navigation solution entrusted with this task now outputs five quantities instead of four: 3-D position and two receiver clock biases. Consequently, at least five observations are necessary to initialize all states, with at least one stemming from each GNSS. To consider the possibility of different receiver clock drifts for GPS and Galileo, a state vector with 19 entries was tested as well (state 19 being the Galileo receiver clock drift). Since the receiver possesses only one common oscillator for all GNSS constellations and the drift between different GNSS time scales is insignificant in comparison, the receiver clock drifts for GPS and Galileo were expected to be indistinguishable. This hypothesis was proven by measurement results, so this 19th state was excluded from further evaluations.

For compatibility with GPS L5-L1 C/A combinations, E5a-E1 combinations are chosen for Galileo preferably. E5b-E1 combinations are the second choice, single-frequency E1 pseudo-ranges the third. The clock correction parameters for E5a-E1 combinations are distributed via Galileo’s *freely-accessible navigation message* (F/NAV). The parameters are also valid for single-frequency E5a processing, but not for E5b-E1 combinations and single-frequency pseudo-ranges on E1 or E5b. In order to process the latter three types of observations correctly, clock correction parameters from Galileo’s *integrity navigation message* (I/NAV) have to be used [16]. Because the clock correction parameters are already referenced to E5a-E1 and E5b-E1 combinations, the formation of these combinations is done according to (6) and no further correction terms are necessary.

(6) $$\rho_{IF}=\frac{\left(\rho_{\alpha }-\gamma_{\beta ,\alpha }\rho_{\beta }\right)}{1-\gamma_{\beta ,\alpha }}$$

Since all Galileo satellites broadcast civil signals on E1, E5a and E5b, only 5% of the processed code observations are not E5a-E1 combinations, compared to 67% of GPS observations that are not L5-L1 C/A. Since the percentage of processed E5b-E1 and E1 Galileo pseudo-ranges is so small, they do not affect the positioning performance substantially. Consequently, I/NAV data and Galileo’s ionospheric correction algorithm for single-frequency users are not included in the algorithm in order to prevent unnecessary complexity. Clock correction parameters and the *Broadcast Group Delay* (BGD) from the F/NAV data as well as the GPS Klobuchar model are used instead. As it does for GPS L5, the JAVAD TRIUMPH-LS does not track the data and pilot components on Galileo E1, E5a and E5b separately. Instead, a combined tracking of E1-B + E1-C is used on E1 and a combined tracking of I + Q components is used on E5a and E5b. The resulting pseudo-ranges receive the observation codes C1X, C5X and C7X in RINEX 3.03, respectively.

## 3. Odometry Model

Even with multi-constellation GNSS, satellite availability is sparse in urban canyons and zero in tunnels. Odometry measurements do not suffer from limited availability due to environmental influences. In modern vehicles equipped with *electronic stability control* (ESC), the *brake control unit* (BCU) receives the rotation increments of all wheels and the steering wheel angle $\psi_{SW}$ from the corresponding sensors. The BCU converts the rotation increments into angular velocities $\omega_{wheel}$ and outputs them onto a data bus, usually a *Controller Area Network* (CAN). For the odometry model, $\omega_{wheel}$ and $\psi_{SW}$ are read from the CAN bus and supplied with a GPS time stamp. The utilization of four different angular wheel velocities as well as the steering wheel angle enables the calculation of a 2-D horizontal velocity for each wheel. In contrast to the commonly used odometry models with just the angular velocity of one undriven, unsteered axle as input, this method works for cornering and slipping vehicles as well. To transform $\omega_{wheel}$ into velocities, the dynamical tire radii $r_{dyn}$ are estimated in the EKF. For this purpose, the EKF’s state vector is augmented by four states, resulting in a total of 22 states (see Table 4). Just as for GNSS processing, the odometry measurements get processed in two steps:

1. Preprocessing: Employing polynomials obtained through calibration for each wheel, the wheel steering angle of each front wheel is computed from the measured steering wheel angle $\psi_{SW}$. Longitudinal and lateral wheel slip are estimated based on the accelerometer outputs of the IMU (corrected for the estimated IMU biases). The horizontal velocity of each wheel is computed from $r_{dyn}$ and $\omega_{wheel}$ as well as longitudinal and lateral slip. For the front wheels, the resulting values have to be rotated into the body coordinate frame via the wheel steering angles. Output values of the odometry preprocessing are the *x*- and *y*-components of the vehicle velocity in the body coordinate frame at each wheel as well as the corresponding covariance matrices.
2. Measurement update: The innovation δz is formed as difference between the measured 2-D velocity at each wheel and its predicted counterpart. These predictions are based on the a-priori estimates of attitude, velocity and dynamical tire radii as well as the vehicle’s geometry, namely the leverarms from the IMU to the four wheels. Finally, δz and the associated measurement noise covariance are used to determine corrections for the state vector’s a-priori estimates. Simultaneously, the state vector’s covariance matrix is updated to reflect the newly incorporated information.

**Figure 1:**
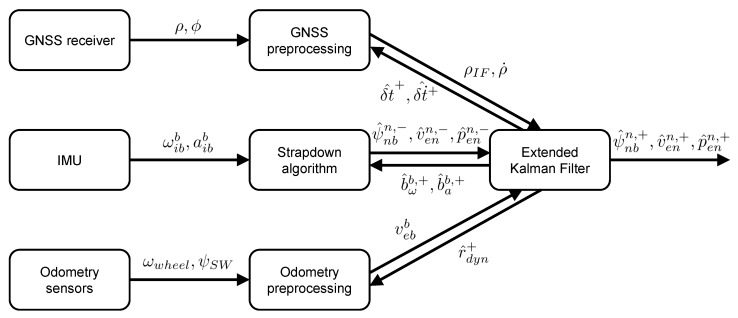
Block diagram of the tightly coupled GNSS/IMU/odometry integration algorithm. Superscript − denotes estimated values before the EKF’s measurement update, superscript + denotes updated estimates after the EKF’s measurement update.

Figure 1 displays a block diagram of the tightly coupled algorithm. Only the most important in- and outputs of each component are shown. The three sensor groups on the left provide the algorithm with raw data: pseudo-ranges ρ and carrier-phase measurements ϕ from the GNSS receiver, 3-D angular velocities $\omega_{ib}^{b}$ and accelerations $a_{ib}^{b}$ from the IMU as well as angular wheel velocities $\omega_{wheel}$ and steering wheel angle $\psi_{SW}$ from the odometry sensors. These inputs are utilized by the preprocessing components in the middle to produce the input data for the EKF: ionosphere-free pseudo-ranges $\rho_{IF}$ and pseudo-range-rates $\dot{\rho }$ from GNSS, a-priori estimates of attitude ${\hat{\psi }}_{nb}^{n,-}$, velocity ${\hat{v}}_{en}^{n,-}$ and position ${\hat{p}}_{en}^{n,-}$ from the strapdown algorithm as well as velocity in the body coordinate frame $v_{eb}^{b}$ for each wheel from odometry. The option to input single-frequency pseudo-ranges into the EKF is omitted in the block diagram for the sake of clarity. After performing the measurement update, the EKF outputs the a-posteriori estimates of attitude ${\hat{\psi }}_{nb}^{n,+}$, velocity ${\hat{v}}_{en}^{n,+}$ and position ${\hat{p}}_{en}^{n,+}$. Additionally, the estimated receiver clock bias ${\hat{\delta t}}^{+}$ and drift ${\hat{\delta \dot{t}}}^{+}$ are fed back to GNSS preprocessing, the estimated IMU biases ${\hat{b}}_{\omega }^{b,+}$ and ${\hat{b}}_{a}^{b,+}$ are fed back to the strapdown algorithm and the estimated wheel radii ${\hat{r}}_{dyn}^{+}$ are fed back to odometry preprocessing.

## 4. Calibration of Differential Code Biases

As discussed in Section 2, the differential code biases (DCBs) prevent the combination of different types of pseudo-range observations without loss of accuracy. To eliminate this restriction, calibration of DCBs is required. For this purpose, the method described in [4] was adopted, albeit on a much smaller scale. A single pseudo-range observation ρ is given by:

(7) $$\rho =R+c\delta t^{rcv}-c\delta t^{sat}+T+I+B^{rcv}-B^{sat}$$

with geometric range *R*, receiver and satellite clock biases δt, tropospheric and ionospheric delays (*T* and *I*) as well as a receiver- and satellite-specific code biases *B*. For two pseudo-range observations to the same satellite at the same epoch, but based on different signals, the terms *R* and *T* are identical. $\delta t^{rcv}$ is identical as well because all differences that might exist in this term are absorbed by the receiver-specific code bias $B^{rcv}$, which differs from signal to signal [4]. The satellite clock bias for a GPS signal α (α = L1 C/A, L2C, L5I or L5Q) is given by (8) [11,12]. The *space vehicle* (SV) clock error $\delta t_{SV}$ is valid for the ionosphere-free combination of L1 P(Y) and L2 P(Y) pseudo-ranges.

(8) $$\delta t_{\alpha }^{sat}=\delta t_{SV}-T_{GD}+{\mathrm{ISC}}_{\alpha }$$

Satellite clock bias corrections for Galileo signals are broadcast via F/NAV for E5a and via I/NAV for E1 and E5b. Since the two different values for $\delta t_{SV}$ are valid for E5a-E1 (F/NAV) and E5b-E1 (I/NAV) ionosphere-free combinations respectively, the corresponding BGD has to be subtracted to get the satellite clock bias for single-frequency pseudo-ranges [16].

(9) $$\begin{array}{cc} \delta t_{E1}^{sat} & =\delta t_{SV}\left(E1,E5b\right)-\mathrm{BGD}\left(E1,E5b\right) \end{array}$$

(10) $$\begin{array}{cc} \delta t_{E5a}^{sat} & =\delta t_{SV}\left(E1,E5a\right)-\gamma_{E1,E5a}\mathrm{BGD}\left(E1,E5a\right) \end{array}$$

(11) $$\begin{array}{cc} \delta t_{E5b}^{sat} & =\delta t_{SV}\left(E1,E5b\right)-\gamma_{E1,E5b}\mathrm{BGD}\left(E1,E5b\right) \end{array}$$

Ionosphere-free linear combinations of pseudo-ranges are formed according to (3) for GPS and according to (6) for Galileo, respectively. Since the DCBs between a ionosphere-free combination (L2C-L1 C/A, L5-L1 C/A, E5a-E1 or E5b-E1) on the one hand and a single-frequency pseudo-range (L1 C/A or E1) on the other hand are to be calibrated, the difference between these two types of observations is formed in (12).

(12) $$\rho_{IF}-\rho_{\alpha }=-c\delta t_{IF}^{sat}+I_{IF}+B_{IF}^{rcv}-B_{IF}^{sat}-\left(-c\delta t_{\alpha }^{sat}+I_{\alpha }+B_{\alpha }^{rcv}-B_{\alpha }^{sat}\right)$$

$I_{IF}$ can be neglected. Assuming that the broadcast values of BGD, ISC and $T_{GD}$ match the satellite-specific code biases well enough that

(13) $$c\delta t_{IF}^{sat}+B_{IF}^{sat}=c\delta t_{\alpha }^{sat}+B_{\alpha }^{sat}=c\delta t_{SV}$$

holds, (12) simplifies to:

(14) $$\begin{array}{cc} \rho_{IF}-\rho_{\alpha }\; & =B_{IF}^{rcv}-B_{\alpha }^{rcv}-I_{\alpha }\\ & ={\mathrm{DCB}}_{IF,\alpha }-I_{\alpha } \end{array}$$

**Figure 2:**
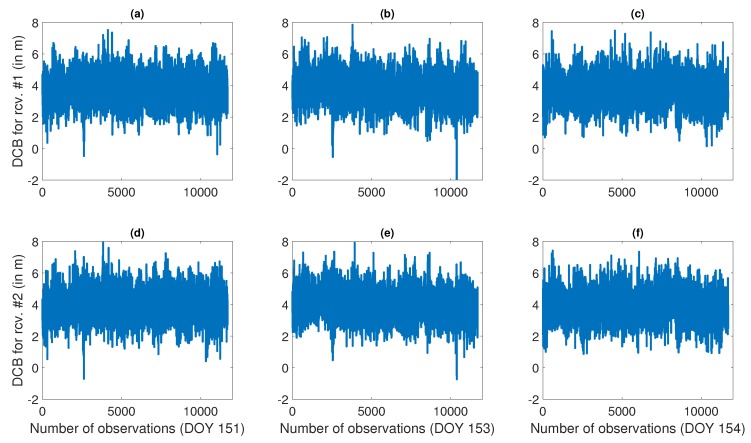
DCB between L2C-L1 C/A ionosphere-free combinations and L1 C/A. Results from three 24 h data sets, each collected with two receivers on days 151 (**a**,**d**), 153 (**b**,**e**) and 154 (**c**,**f**) of 2018.

In order to calibrate ${\mathrm{DCB}}_{IF,\alpha }$, the DCB between a ionosphere-free linear combination $\rho_{IF}$ and a single-frequency pseudo-range $\rho_{\alpha }$, the ionospheric delay for the signal α needs to be eliminated. This was done with *total electron content* (TEC) maps provided by the *International GNSS Service* (IGS) via the *Ionosphere Map Exchange Format* (IONEX). Input data for DCB calibration was collected with two JAVAD TRIUMPH-LS receivers, both connected to the same roof antenna. In accordance to [4], 24 h data sets with 30 s sampling rate and a minimum elevation of 20${}^{\circ }$ were used. Data collection was performed on days 151, 153 and 154 of 2018. On day 152, both receivers were shut down and restarted to enable the detection of potential turn-on biases. Figure 2 displays the estimated DCBs between L2C-L1 C/A ionosphere-free combinations and L1 C/A for the six data sets. Every day a total of approximately 12,000 observations was collected with each receiver. Although the DCB estimates are noisy, it is obvious that the DCB is nonzero. No significant variation over time is evident. Also, no turn-on bias can be identified because the curves for *day of year* (DOY) 151 (Figure 2a,d) are similar to the ones from DOY 153 (Figure 2b,e) and DOY 154 (Figure 2c,f).

These findings are supported by the statistical analysis presented in Table 5: All four calibrated DCBs are stable over the examined time span. The standard deviation for a single 24 h set ranges from approximately 0.6 m to approximately 0.8 m while the maximum difference of the means is approximately 0.3 m. For the combination of all six data sets, the standard deviation ranges from approximately 0.7 m to approximately 0.8 m. The means estimated from all six data sets combined reveal the reason why the proposed processing of different types of ionosphere-free combinations together with single-frequency observations for GPS (see Section 2.2) did not work properly: While the DCB of −1.9 m between L5-L1 C/A and L1 C/A is small enough to still maintain some benefit from the utilization of dual-frequency observations, this is apparently not the case for the DCB of 3.8 m between L2C-L1 C/A and L1 C/A. The combined processing of all three observation types (L1 C/A, L2C-L1 C/A and L5-L1 C/A) amplifies the problem because the DCB between L2C-L1 C/A and L5-L1 C/A is approximately 5.7 m. The problem is less pronounced on Galileo: While the DCBs between E5a-E1 and E1/E5b-E1 and E1/E5a-E1 and E5b-E1 are approximately −3.0 m/3.3 m/−6.3 m respectively, the fact that all Galileo satellites broadcast the same signals results in a high availability of the preferred E5a-E1 ionosphere-free combinations. That is why only very few E5b-E1 combinations and E1 single-frequency pseudo-ranges have to be used as backup. The common-mode error of −3.0 m on all E5a-E1 ionosphere-free combinations is simply added to the receiver clock bias $c\delta t^{rcv}$ and does not affect positioning performance.

With the obtained calibration values for all four types of ionosphere-free combinations, it is possible to use a total of six different types of pseudo-range observations simultaneously: L5-L1 C/A and E5a-E1 as first choice, L2C-L1 C/A and E5b-E1 as second choice, single-frequency L1 C/A and E1 as third choice for GPS and Galileo, respectively. To verify the DCB calibration and provide an impression about the achievable accuracy for the upcoming Section 5, single-epoch solutions were computed from the six collected 24 h data sets, making use of all six different types of pseudo-range observations. This was done in three different ways: once with our self-developed software and the calibrated DCBs, once with our self-developed software but without the calibrated DCBs and once with RTKLIB [17], using similar settings as in the self-developed software (positioning mode: single, frequency bands: L1 + L2 + L5, filter type: forward, elevation mask: 5${}^{\circ }$, ionosphere correction: IF combinations, ephemeris/clock: broadcast, GPS + Galileo). While the frequency bands and constellations to be used in processing the input data can be selected in RTKLIB, there is no record in the output data specifying exactly which observations have been used in a certain epoch. The obtained position was compared to the known position of a roof antenna (standard deviation of antenna position below 3 cm in each coordinate) to form the RMS errors given in Table 6. Calibration of the DCBs practically cuts the RMS error for each coordinate in half, resulting in an overall RMS error of [0.6;0.8;1.5] m compared to [1.2;1.5;2.9] m with uncalibrated DCBs. RTKLIB achieves an intermediate accuracy with an overall RMS error of [0.8;1.1;2.1] m. These results verify the DCB calibration and support the approach to work with different types of pseudo-range observations simultaneously.

## 5. Localization Algorithm Performance

In order to assess the performance of the upgraded localization algorithm, two sets of measurement data were collected and evaluated. GNSS input data for the integration algorithm were recorded with a JAVAD TRIUMPH-LS, operating at 5 Hz. IMU input data for the integration algorithm were recorded with an Xsens MTi-G-700, operating at 200 Hz. Odometry input data for the integration algorithm were recorded from the vehicle’s CAN bus with 100 Hz.

A reference solution was computed with Novatel’s Waypoint-Inertial Explorer software, using multi-frequency GNSS data from two JAVAD TRIUMPH-LS (RTK with base and rover at 5 Hz) and inertial data from a navigation grade IMU (iMAR iNAV-RQH-1003, operating at 300 Hz).

The two sets of measurement data are:

1. Roughly 40 min long drive over a distance of approximately 13 km on the 14th of March 2018 through the inner city of Darmstadt, including a passage through a tunnel lasting roughly 40 s/450 m.
2. Roughly 100 min long drive over a distance of approximately 90 km on the 14th of March 2018, including towns with multi-story buildings on both sides of the road, villages with smaller houses, country roads (with and without forest), freeways and a passage through a tunnel lasting roughly 55 s/1000 m.

**Figure 3:**
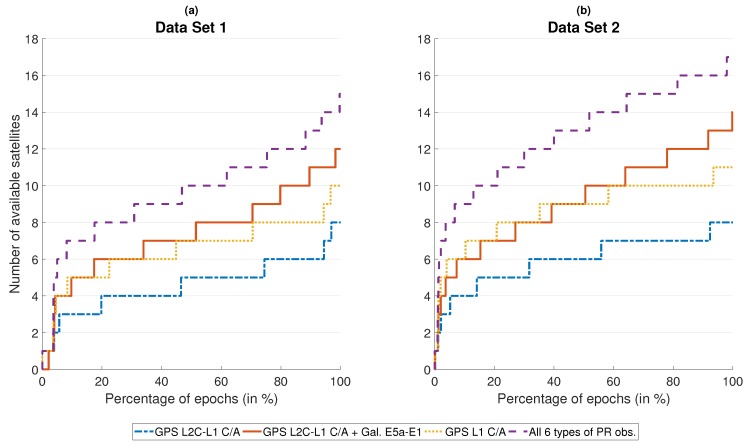
Percentage of GNSS measurement epochs in which no more than the indicated number of satellites was available.

**Figure 4:**
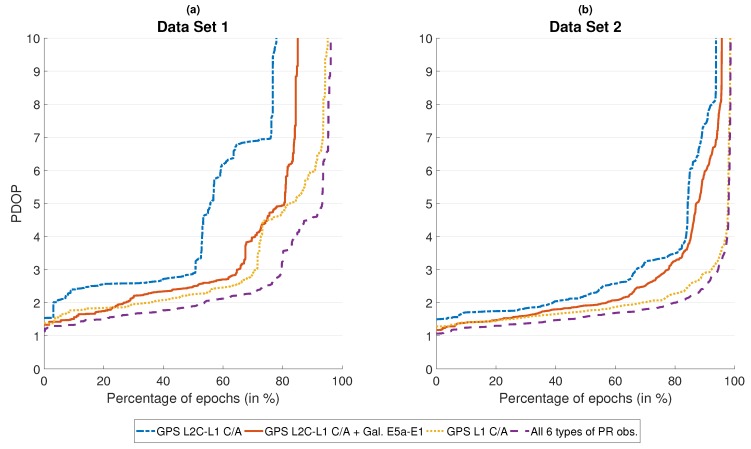
Percentage of GNSS measurement epochs in which PDOP was at least as good as indicated.

Figure 3 and Figure 4 present information about the GNSS availability for both data sets. In both figures, satellites with an elevation smaller than 5${}^{\circ }$ are neglected. Low-elevation satellites, which typically experience larger multipath errors, do not impact the solution as much as high-elevation satellites due to the elevation-dependent weighting according to (4). Figure 3 shows the number of received code observations for GPS L1 C/A, L2C-L1 C/A combinations, the sum of L2C-L1 C/A and E5a-E1 combinations as well as the sum of all six pseudo-range observations together. Figure 4 quantifies the quality of the satellite geometry with the corresponding *position dilution of precision* (PDOP). The curves correspond to different stages of algorithm development: single-frequency GPS, multi-frequency GPS, multi-frequency/multi-constellation GNSS without calibrated DCBs and finally multi-frequency/multi-constellation GNSS with calibrated DCBs. The L2C-L1 C/A ionosphere-free combination was chosen for GPS since it is broadcast by more satellites than L5-L1 C/A. So, without information about the magnitude of receiver DCBs, working with L2C-L1 C/A combinations as the only GPS code observation offers the highest satellite availability of all civil multi-frequency GPS signals.

As Figure 3 demonstrates, data set 1 suffers from reduced satellite visibility compared to set 2 due to the surrounding buildings. For data set 1 (Figure 3a), single-frequency GPS L1 C/A pseudo-ranges from at least four satellites are available more than 90% of the time, while at least eight satellites are available more than 30% of the time. A maximum ten satellites are received simultaneously. In the second data set (Figure 3b), these numbers increase to more than 90%, more than 75% and a maximum of eleven, respectively. The number of available L2-L1 C/A combinations is on average two lower than for L1 C/A in the first data set and three lower than for L1 C/A in the second data set. Compared to GPS L1 C/A, the sum of available L2C-L1 C/A and E5a-E1 combinations is on average slightly higher in both data sets. This corresponds to the total number of healthy satellites in orbit: 31 for GPS L1 C/A and 33 for L2C-L1 C/A + E5a-E1 (19 GPS + 14 Galileo) [10,18]. However, the combined processing of GPS and Galileo introduces another unknown (two receiver clock biases instead of one). Therefore, the number of available pseudo-range observations for position determination is still slightly lower for L2C-L1 C/A + E5a-E1 than it is for GPS L1 C/A. The best results occur for joint processing of all six types of pseudo-range observations: even in data set 1 with its urban canyons and tunnel, code observations from ten or more different satellites are available more than 50% of the time with a maximum of 15. For data set 2, this percentage rises to 80% with a maximum of 17. This demonstrates the advantages of the approach to work with L5-L1 C/A and E5a-E1 ionosphere-free combinations preferably, but use L2C-L1 C/A and E5b-E1 combinations as well as L1 C/A and E1 single-frequency pseudo-ranges as backup whenever observations on L5/E5a are unavailable. The advantage is more pronounced on GPS than on Galileo since all Galileo satellites broadcast E5a signals while currently only 12 out of 31 GPS satellites broadcast L5 signals [10].

Figure 4 displays how the varying number of simultaneously available pseudo-ranges affects the PDOP. PDOP is calculated from all pseudo-ranges that were used in the EKF’s measurement update, i.e., all pseudo-ranges that passed the plausibility check. Single-frequency GPS achieves a PDOP of less than four in approximately 73% of epochs in data set 1 (Figure 4a) and in approximately 97% of epochs in data set 2 (Figure 4b). For L2C-L1 C/A combinations, these numbers drop to 53% and 84%, respectively. The inclusion of E5a-E1 pseudo-ranges reduces the PDOP again, but it is still higher than for single-frequency GPS: A PDOP less than four is achieved in 71%/85% of epochs for the two data sets. The utilization of all six types of pseudo-range observations offers the best signal geometry in both data sets. This effect is more pronounced in data set 1, where a PDOP less than four is attained in approximately 84% of epochs, proving that multi-constellation GNSS is most beneficial in situations with a high amount of signal blockage. For data set 2, a PDOP less than four is achieved in approximately 98% of epochs, offering no significant benefit in comparison to single-frequency GPS. Looking at the complete graphs emphasizes the benefits of being able to process all types of pseudo-ranges simultaneously: When compared to the results obtained with L2C-L1 C/A and E5a-E1 observations, the PDOP is lower in all epochs. The largest improvement is observed in the right third of each plot, corresponding to epochs with a relatively low number of available L2C-L1 C/A and E5a-E1 observations.

The following subsections deal with estimation accuracy. Accuracy is quantified by the estimation error $\tilde{x}$ and its statistical distribution. $\tilde{x}$ is defined as the difference between the value $\hat{x}$, estimated by the localization algorithm being evaluated, and the reference value $x_{ref}$.

(15) $$\tilde{x}\left(k\right)=\hat{x}\left(k\right)-x_{ref}\left(k\right)$$

*k* is an arbitrary point in time and takes on values between 1 and $N_{j}\in N$ during one data set *j*. The reference value is provided by the reference trajectory. Depending on the type of quantity being evaluated, $\tilde{x}$ can be a scalar or a vector. In both cases, the error is a random variable and fully characterized by its *cumulative distribution function* (CDF) [19]. Section 5.1 focuses on the accuracy of attitude and velocity estimation. Because the main improvement in these quantities compared to the basic GPS L1 algorithm without odometry stems from odometry measurements, the multi-frequency/multi-constellation algorithm with odometry turned off is compared to the same algorithm, but with odometry turned on. Section 5.2 focuses on the accuracy of position estimation. Because the main improvement in the determination of absolute position compared to the basic GPS L1 algorithm stems from the inclusion of multi-frequency/multi-constellation pseudo-ranges, the three different stages of algorithm development (see Section 2.1, Section 2.2 and Section 2.3) are compared to each other. To illustrate the improvement in positioning accuracy provided by odometry measurements in GNSS-denied environments, the position error when driving through tunnels with and without odometry aiding is presented as well.

### 5.1. Attitude and Velocity Accuracy

While pseudo-ranges are mainly responsible for position aiding, attitude and velocity estimates derived from IMU measurements are mainly corrected by pseudo-range-rate and odometry observations. Pseudo-range-rates are measured and applied in the navigation coordinate frame, whereas the wheel velocities are measured in the body coordinate frame and applied in the navigation frame. Consequently, the inclusion of odometry measurements improves the estimation accuracy of yaw angle and velocity in the body coordinate frame. During periods with poor or no GNSS reception, odometry aiding improves estimation accuracy of velocity in the navigation coordinate frame and thereby position accuracy, as long as yaw angle accuracy is sufficiently high.

**Figure 5:**
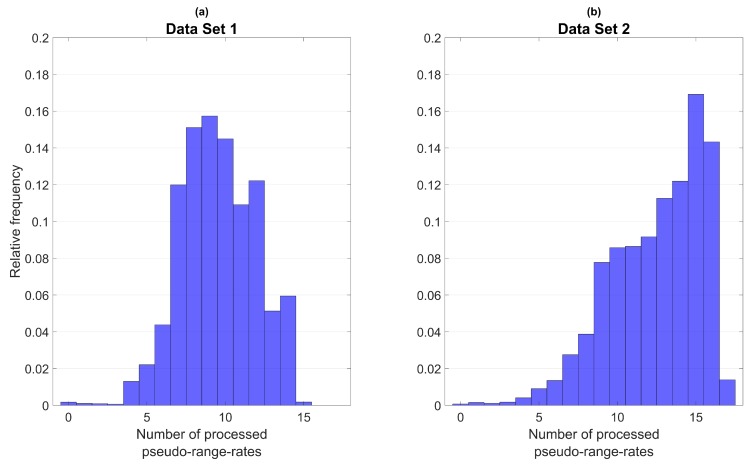
Relative frequency distribution for the number of processed pseudo-range-rates. Multi-frequency/multi-constellation algorithm.

To provide information about the availability of pseudo-range-rate observations during the test scenarios, Figure 5 shows the relative frequency distribution of the number of processed pseudo-range-rates for the multi-constellation algorithm in both data sets. Data set 1 (Figure 5a) has on average 9.5, data set 2 (Figure 5b) on average 12.5 observations available. For data set 1, six or more pseudo-range-rates are processed in more than 95% of epochs. In data set 2, it is eight or more observations in more than 95% of epochs. These high availability levels also show in the numerical analysis presented in Table 7: yaw error RMS is less than 1${}^{\circ }$ in both sets, with and without odometry. The RMS error of $v_{en}^{n}$ is less than 10 $\frac{\mathrm{cm}}{s}$ in each coordinate. When comparing the results for odometry turned on and odometry turned off to each other, two main improvements can be identified when odometry is turned on: The 95% quantile of the yaw error gets reduced and horizontal accuracy of velocity estimation in the body coordinate frame is improved, especially along the lateral (*y*-)axis. The fact that odometry mainly benefits yaw angle estimation by reducing its 95% quantile can be explained as follows: Whenever the vehicle is traveling in a straight line, the yaw angle can be estimated reliably based on GNSS because the direction of travel is identical with the *x*-axis of the body coordinate frame. This is true most of the time. But whenever the vehicle is turning, the direction of travel and the body *x*-axis do not coincide anymore, causing the yaw angle estimate from GNSS/IMU integration to be less accurate. The odometry model is still able to estimate the velocity in the body coordinate frame reliably even during turns, thus improving yaw angle estimation accuracy in these types of situations. The same explanation holds for the accuracy of lateral velocity estimation: When driving in a straight line, $v_{y}$ is zero and can be estimated accurately with GNSS. But during turns, $v_{y}$ is nonzero and the inclusion of measurements derived from the odometry model improves its estimation accuracy.

### 5.2. Position Accuracy

**Figure 6:**
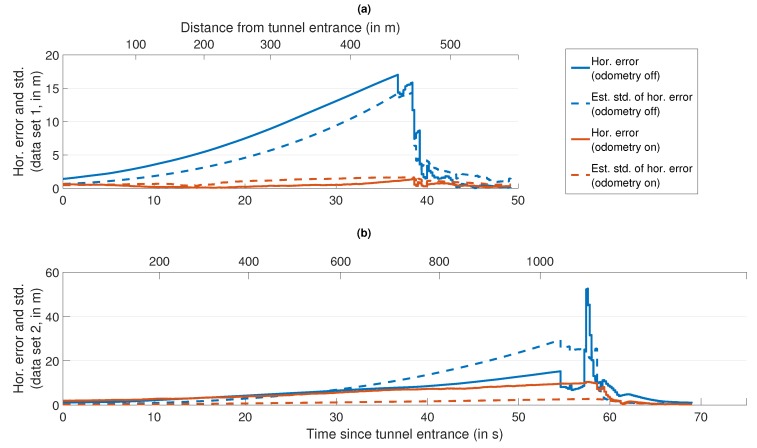
Horizontal position error and its estimated standard deviation during passages through different tunnels. Multi-frequency/multi-constellation algorithm, with and without odometry aiding. (**a**) Tunnel in data set 1. (**b**) Tunnel in data set 2. Note the different scaling of the two vertical axes.

This subsection deals with the position accuracy achieved by the different algorithm versions over the course of the two data sets. In the beginning, Table 8 gives an overview of the most important accuracy metrics, evaluated for all three algorithm versions (single-frequency GPS, multi-frequency GPS and multi-frequency/multi-constellation GNSS) with both odometry turned off and on. As our previous research [20] has shown, the evaluation of these metrics over a whole data set combined can only provide a first impression of the achievable accuracy, but is insufficient for a detailed analysis. That is why Table 10 breaks down the RMS position error for the multi-frequency/multi-constellation algorithm with odometry aiding in six categories, depending on the reference solution quality. The subsection concludes with an evaluation of the horizontal position error and its estimated standard deviation during the passages through tunnels in Figure 6 to emphasize the importance of odometry aiding in GNSS-denied environments. All results in this subsection were obtained with the calibrated DCBs, using all types of pseudo-range observations available to the respective algorithm version simultaneously.

The results in Table 8 show that the multi-frequency/multi-constellation algorithm is capable of improving all major position accuracy metrics. The arithmetic mean is given to demonstrate that position estimation with multi-frequency GNSS is less biased than with single-frequency observations. This is especially obvious when looking at the vertical coordinate’s mean in data set 2: Without odometry aiding, it is −1.8 m for single-frequency GPS, −0.3 m for multi-frequency GPS and −0.5 m for multi-frequency/multi-constellation GNSS. The reduction in the mean’s absolute value when including GPS pseudo-ranges on multiple frequencies confirms the ability of multi-frequency measurements to eliminate the ionospheric error, improving unbiasedness primarily in the vertical coordinate. When quantifying estimation accuracy in comparison to an external reference solution as is done here, the RMS error is more suitable than the error’s standard deviation. While the standard deviation quantifies the variation around the mean, the RMS error quantifies the variation around the reference solution, delivering a metric that quantifies both precision and unbiasedness combined. In terms of the RMS error, the multi-frequency/multi-constellation algorithm delivers the best performance for all coordinates in each column. The horizontal position error quantiles in the last row of Table 8 are important because automotive positioning is mainly concerned with horizontal accuracy. Since all vehicles travel on the road, vertical accuracy is not as critical. The multi-frequency/multi-constellation algorithm without odometry aiding achieves the best performance in the 50% quantiles. When odometry is included, the 50% quantiles rise slightly (by 0.1 m in each data set). The information about the vehicle’s velocity provided by the odometry leads to a decreased estimated covariance of the position error (via covariance propagation through the EKF’s system model). That is why the pseudo-ranges receive relatively smaller weights when odometry aiding is turned on in comparison to when odometry aiding is turned off. As a result, position errors will grow slower when GNSS is unavailable (see Figure 6), but may also decrease slower when GNSS is available. This circumstance improves positioning performance when GNSS reception is limited or GNSS measurements are faulty, but can degrade positioning performance slightly when GNSS reception changes from poor to good. This is a consequence of the relative magnitude of variances in the measurements’ stochastic model. Further optimization of these variances is necessary to prevent this undesired behavior. An example for the improvement in position accuracy provided by the odometry is the reduction of the 95% quantile for data set 1 from 6.2 m without odometry aiding to 4.2 m with odometry aiding for the multi-frequency/multi-constellation algorithm. For the multi-frequency GPS algorithm, the improvement is even larger, reducing the 95% quantile for data set 1 from 8.2 m to 4.4 m. These findings correspond to the hypothesis that odometry aiding is most beneficial to positioning accuracy when GNSS measurement quality is poor, which is the case for a significant amount of time in data set 1 (see Table 10 for a more detailed analysis of GNSS measurement quality in both data sets).

A distinction between an error in the output of the localization algorithm under test $\hat{p}$ and an error in the reference value $p_{ref}$ is generally impossible. Consequently, the evaluation of the estimation error ${\tilde{p}}_{en}^{n}$ is not as meaningful when the reference solution’s quality is poor as it is when this quality is high. That is why the data sets need to be dissected into multiple parts to facilitate a more detailed analysis of the algorithm’s positioning performance. Dissecting is done based on the quality of the reference solution. Table 9 lists Inertial Explorer’s six different *quality numbers* as well as their description and the corresponding 3-D accuracy. Based on the 3-D accuracy estimated by the processing software, Inertial Explorer assigns one of these quality numbers to each epoch of the reference solution.

On the basis of the RMS error obtained from the static 24 h data sets (see Table 6), a 3-D accuracy in the order of 1–2 m is expected from the multi-frequency/multi-constellation algorithm under good conditions. Therefore, only epochs with quality numbers 1 and 2 (max. estimated 3-D error 0.4 m) are able to provide a reference solution that is significantly more accurate than the algorithm under test. Epochs with quality numbers 3 and 4 yield a maximum estimated 3-D error of 2 m, putting them on par with the RMS error obtained from the static 24 h sets. Epochs with quality numbers 5 and 6 may produce 3-D errors up to 10 m, rendering them unfeasible as reliable reference. Because both the reference and the algorithm under test suffer from the same limitations in GNSS signal reception and thus GNSS measurement quality, the algorithm under test is likely to produce worse results during these epochs than it produces under the very good conditions prevalent in the static data. Hence, the reference is expected to be more accurate than the algorithm under test during epochs with quality numbers 3 and 4 as well. However, as the magnitude of the estimation error is comparatively more uncertain, the RMS errors for these quality numbers given in Table 10 could be significantly higher or lower. For epochs with quality numbers 5 and 6, it is unclear which solution is more accurate: On the one hand, the reference solution encompasses GNSS correction data from a base station as well as measurements from a navigation grade IMU. On the other hand, the algorithm under test includes odometry measurements and the poor quality number already suggests that GNSS reception is seriously impaired, reducing the usefulness of the GNSS correction data used in the reference solution. That is why the RMS errors for these quality numbers in Table 10 can only give a rough impression of the algorithm’s performance under these conditions.

Table 10 details the performance of the most sophisticated algorithm version (multi-frequency/multi-constellation with calibrated DCBs and odometry aiding) depending on the reference solution quality. The different characteristics of the two data sets are represented in the percentage of epochs for each quality number: While 60% of epochs in data set 1 offer high accuracy (quality number 1 or 2), 34% feature intermediate accuracy (quality number 3 or 4) and 6% exhibit poor accuracy (quality number 5 or 6), these numbers are 70%, 27% and 3% in data set 2. This confirms that data set 2 comprises better GNSS reception conditions than data set 1 on average. Looking at the epochs with a reference solution quality number of 1 or 2, the RMS error in the horizontal coordinates is between 0.7 m and 1.1 m, compared to values between 0.6 m and 0.8 m for the individual static 24 h sets. The fusion of sensor data from GNSS, IMU and odometry is therefore capable of determining antenna position and attitude under kinematic conditions well enough to provide an estimate of the vehicle’s position with similar accuracy as for static measurements with very good GNSS reception. The same holds for the vertical RMS error in data set 2, which is 1.3 m/1.4 m for a reference solution quality number of 1/2, compared to values between 1.3 m and 1.7 m for the individual static 24 h sets. In data set 1, the vertical RMS error is significantly higher with 2.4 m/2.5 m for a reference solution quality number of 1/2. This stems from an interval of approximately 5 min spend uninterruptedly inside an urban canyon. During this time span, the vertical position error rises to over 7 m. Odometry cannot provide information about the vehicle’s vertical velocity and is therefore unable to aid the vertical position. The horizontal position error does not exceed 3 m throughout the same interval as odometry is able to limit the horizontal velocity error. The reference solution mostly achieves quality number of 1 or 2 while inside the urban canyon. This is not the case when GNSS observations of base and rover are processed without measurements from the navigation grade IMU, demonstrating that GNSS conditions are in fact very challenging. In both data sets, the RMS position error for the epochs with reference solution quality number 3 is roughly on the same level as it is for the quality numbers 1 and 2. The single exception is the north component in data set 1 with an RMS error of 2.1 m. The fusion of GNSS, IMU and odometry data is therefore able to extent the range of high accuracy from epochs with good GNSS reception conditions to epochs when GNSS conditions start to deteriorate. Starting with reference solution quality number 4, the RMS position error in both data sets rises significantly. The maximum 3-D position error in data set 1 is approximately 20 m, in data set 2 approximately 10 m. Both error maxima occur for a reference solution quality number of 6. Hence, the numerical values can only serve as coarse assessment of the actual error since the quality of the reference solution is substantially impaired. In data set 2, the maximum 3-D position error originates from driving through a tunnel. The two tunnel passages (one in each data set) are examined more closely in the following paragraph.

Figure 6 demonstrates the benefits of odometry aiding on the position error in GNSS-denied environments such as tunnels. In data set 1 (Figure 6a), the horizontal position error without odometry aiding grows to approximately 17 m and its estimated standard deviation to approximately 15 m inside the tunnel. Once the first GNSS pseudo-range observations are received again (roughly 38 s/450 m after the tunnel entrance), the error and its estimated standard deviation quickly fall to the same levels as before the tunnel. When odometry measurements are included, the maximum horizontal position error is approximately 1.5 m and the maximum value of the estimated standard deviation is approximately 1.6 m. Both with and without odometry, the estimated standard deviations correlate very well with the actual errors. It should be noted that this tunnel features a 90${}^{\circ }$ left-hand turn between 30 s and 35 s and is different from the tunnel in data set 2, which is longer (approximately 1000 m) and consists of a series of elongated left- and right-hand turns. The differences in the actual error during the passage through that second tunnel (Figure 6b, 0–55 s/0–1000 m) are not as large: 15 m without odometry compared to 10 m with odometry. The estimated standard deviation rises to approximately 29 m without the inclusion of odometry measurements and approximately 3 m with odometry aiding. However, a few seconds after the end of the tunnel, the horizontal position error peaks up to more than 50 m without odometry aiding before it sinks to pre-tunnel levels. This is due to erroneous pseudo-range observations. Since the estimated horizontal position standard deviation is approximately 25 m at the time, the signal plausibility check operating at the 3σ-level cannot identify the incorrect measurements. When odometry is turned on, the estimated horizontal standard deviation is sufficiently low (approximately 3 m) to be able to exclude the erroneous pseudo-range observations, resulting in a maximum error of just approximately 10 m.

## 6. Conclusions

This paper describes a way to incorporate observations from multiple GNSS constellations and on multiple carrier frequencies as well as odometry measurements into a tightly coupled GNSS/IMU/odometry integration algorithm. An existing algorithm working with GPS L1 C/A code pseudo-ranges and time-differenced carrier-phase observations as the only GNSS data is upgraded to include code pseudo-range measurements on GPS L2C and GPS L5 in a first step. The pseudo-ranges on different carrier frequencies are used to form ionosphere-free linear combinations. These combinations directly cancel out most of the ionospheric error and can be processed without having to change the EKF’s state vector. The increased code tracking noise of ionosphere-free combinations is filtered out due to the EKF’s inherent smoothing capability and does not influence positioning performance negatively. Initially, the attempt to work with L2C-L1 C/A and L5-L1 C/A ionosphere-free combinations as well as single-frequency L1 C/A pseudo-ranges simultaneously did not accomplish the expected gain in accuracy. On the contrary, positioning performance became worse than for the basic single-frequency algorithm. Large DCBs, especially for the L2C-L1 C/A combinations, were suspected to be the cause of this behavior. A subsequent calibration of DCBs for GPS and Galileo verified this hypothesis and asserted the long-term stability of all relevant DCBs, enabling the utilization of a total of six different types of pseudo-range observations (three for GPS, three for Galileo) within the same integration filter. The inclusion of Galileo observations introduces two additional types of biases: the GGTO and an ISB. While the former could be compensated via information from one of the navigation messages, the latter proved to be too large to be ignored. As these two biases influence the observations in the same way, they can be subsumed into one bias term. To absorb the effects of this bias term, an additional state for the Galileo receiver clock bias was added to the EKF’s state vector.

In an effort to facilitate the correction of the navigation solution derived from IMU measurements even in situations without GNSS reception, odometry observations were introduced into the localization algorithm as additional source of information. For this purpose, an odometry model with five inputs (four angular wheel velocities and the steering wheel angle) was incorporated. This model outputs the 2-D horizontal velocity for each wheel and provides reliable velocity observations even for cornering and slipping vehicles.

The evaluation of two data sets proves the advantages of including multi-frequency/multi-constellation GNSS observations and odometry measurements into the localization algorithm. Incorporation of Galileo satellites increases the number of simultaneously available measurements and reduces the PDOP. This is especially true in situations with limited GNSS availability, e.g. inside the urban canyons contained in the first data set. The utilization of ionosphere-free combinations reduces the RMS position error, especially during periods of favorable GNSS reception conditions. Due to the successful calibration of DCBs, the localization algorithm is capable of working with the maximum possible amount of ionosphere-free combinations at any time, reducing the impact of ionospheric errors to a minimum. In terms of odometry, the evaluation points out two main improvements accomplished by the odometry model: On the one hand, the inclusion of odometry measurements enhances the estimation accuracy of yaw angle and lateral velocity, primarily when the vehicle is cornering. On the other hand, odometry aiding is able to maintain the horizontal position accuracy achieved during periods of unimpaired GNSS reception throughout periods with poor or no GNSS reception. This reduces the growth of the horizontal position error over time and results e.g. in a significant decrease of the horizontal 95% error quantile. Additionally, the estimated standard deviation of the position error is kept comparatively low during GNSS outages, making the position estimation less vulnerable to multipath once GNSS reception is possible again.

In order to get plausible values for these estimated standard deviations, the stochastic models of all input data has to match the actual measurement errors as closely as possible. In addition, this will assure the correct weighting of all input data relative to one another. While the measurement noise variance model for GNSS observations presented in Section 2.2 does produce good results in the evaluated data sets, it is not based on a statistical analysis of actual measurement errors. The same holds for the stochastic model of the odometry data, which has not been discussed in this paper. We plan to conduct a thorough analysis of the actual measurement errors for both GNSS and odometry input data with the purpose of developing more realistic stochastic models for the input data. Based on these models it is also possible to implement a more sophisticated method for detection and exclusion of erroneous observations, e.g., due to multipath for GNSS and due to tire slip for odometry. In the case of GNSS, this detection can be facilitated by the fact that some observations remain unused in the current algorithm design: If pseudo-ranges on all three frequencies are available, the ones on L2C (for GPS) and E5b (for Galileo) are not incorporated into the ionosphere-free combination. Consequently, they provide an independent source of information which can be beneficial to outlier detection. The goal of these prospective research areas is to make the algorithm more robust against measurement errors and to enable a continuous quality monitoring in all processing steps from input data to navigation solution.

## Figures and Tables

**Table 1:** State Vector of the Basic Algorithm.

State	Description	Symbol
1–3	Attitude error	$\Delta \psi_{nb}^{n}$
4–6	Velocity error	$\Delta v_{en}^{n}$
7–9	Position error	$\Delta p_{en}^{n}$
10–12	Gyroscope offset error	$\Delta b_{\omega }^{b}$
13–15	Accelerometer offset error	$\Delta b_{a}^{b}$
16	Error of GPS receiver clock bias	Δcδt
17	Error of GPS receiver clock drift	$\Delta c\delta \dot{t}$

**Table 2:** Differences in Estimated Receiver Clock Bias (Compared to Single-Frequency L1 C/A Processing) and Positioning Performance for Three Sets of Kinematic Measurement Data. N/A Indicates it was not Possible to Compute a Solution due to a Lack of Available GPS Block IIF Satellites.

Processed Code Observations	Mean and Std. of Difference (in m)	Positioning Performance (Ranked)
L1 C/A	0/0/0	2/2/4
	0/0/0	
L2C(M + L)-L1 C/A	−6.5/−7.5/−6.9	3/3/2
	1.5/ 1.8/ 2.4	
L2C(M + L)-L1 C/A	−1.5/ 0.7/−4.3	4/5/1
> L1 C/A	1.7/ 1.7/ 2.1	
L5(I + Q)-L1 C/A	1.6/N/A/N/A	7/N/A/N/A
	11.9/N/A/N/A	
L5(I + Q)-L1 C/A	−0.7/−0.5/−0.6	1/1/2
> L1 C/A	0.7/ 0.5/ 0.6	
L5(I + Q)-L1 C/A	3.0/−8.4/−7.4	6/6/6
> L2C(M + L)-L1 C/A	8.1/ 7.2/ 2.8	
L5(I + Q)-L1 C/A	0.3/−0.6/−5.3 2.0/ 1.5/ 2.1	
> L2C(M + L)-L1 C/A		5/4/5
> L1 C/A		

**Table 3:** Receiver Clock States of the Multi-GNSS Algorithm.

State	Description	Symbol
16	Error of GPS receiver clock bias	$\Delta c\delta t_{GPS}$
17	Error of GNSS receiver clock drift	$\Delta c\delta {\dot{t}}_{GNSS}$
18	Error of Gal. receiver clock bias	$\Delta c\delta t_{Gal}$

**Table 4:** Additional States for the Odometry Model.

State	Description	Symbol
19	Dyn. tire radius error (front left)	$\Delta r_{dyn,FL}$
20	Dyn. tire radius error (front right)	$\Delta r_{dyn,FR}$
21	Dyn. tire radius error (rear left)	$\Delta r_{dyn,RL}$
22	Dyn. tire radius error (rear right)	$\Delta r_{dyn,RR}$

**Table 5:** Mean (First Line of Each Cell) and Standard Deviation (Second Line of Each Cell) of Estimated DCBs. Results from Three 24 h Data Sets, Each Collected with Two Receivers on Days 151, 153 and 154 of 2018.

Receiver	DCB Between L2C-L1 C/A and L1 C/A (in m)	DCB Between L5-L1 C/A and L1 C/A (in m)	DCB Between E5a-E1 and E1 (in m)	DCB Between E5b-E1 and E1 (in m)
JAVAD	3.7/3.8/3.7	−2.0/−2.0/−2.0	−3.1/−3.0/−3.0	3.4/3.2/3.2
TRIUMPH-LS #1	0.8/0.8/0.8	0.7/ 0.6/ 0.7	0.7/ 0.6/ 0.7	0.7/0.8/0.8
JAVAD	3.9/4.0/3.9	−1.8/−1.9/−1.8	−2.8/−2.9/−2.9	3.5/3.3/3.3
TRIUMPH-LS #2	0.8/0.8/0.8	0.7/ 0.6/ 0.7	0.7/ 0.6/ 0.7	0.7/0.8/0.8
#1 and #2	3.8	−1.9	−3.0	3.3
combined	0.8	0.7	0.7	0.8

**Table 6:** RMS Position Error for Single-Epoch Solutions. Results from Three Static 24 h Data Sets, Each Collected with Two Receivers on Days 151 (First Line of Each Cell), 153 (Second Line of Each Cell) and 154 (Third Line of Each Cell) of 2018. ENU Coordinate Frame.

Receiver	Single-Epoch Solutions, Computed Including the Calibrated DCBs (in m)	Single-Epoch Solutions, Computed Without the Calibrated DCBs (in m)	Single-Epoch Solutions, Computed by RTKLIB with IF Combinations (in m)
JAVAD TRIUMPH-LS #1	[0.7;−0.8;−1.6]	[1.3;−1.5;−3.0]	[0.9;−1.2;−2.1]
	[0.6;−0.8;−1.4]	[1.0;−1.5;−2.6]	[0.8;−1.0;−2.0]
	[0.6;−0.7;−1.4]	[1.3;−1.5;−2.9]	[0.8;−1.1;−2.2]
JAVAD TRIUMPH-LS #2	[0.7;−0.8;−1.7]	[1.3;−1.4;−3.0]	[0.9;−1.3;−2.1]
	[0.6;−0.8;−1.4]	[1.1;−1.6;−2.8]	[0.8;−0.9;−2.2]
	[0.6;−0.7;−1.3]	[1.2;−1.6;−2.9]	[0.8;−1.1;−2.2]
All six data sets combined	[0.6;−0.8;−1.5]	[1.2;−1.5;−2.9]	[0.8;−1.1;−2.1]

**Table 7:** Accuracy Metrics for Attitude (Roll; Pitch; Yaw) and Velocity (FLU and ENU Coordinate Frames) Error. Multi-Frequency/Multi-Constellation Algorithm, with and Without Odometry Aiding.

Quantity	Unit	Data Set 1,	Data Set 1,	Data Set 2,	Data Set 2,
		Odometry Off	Odometry On	Odometry Off	Odometry On
RMS of ${\tilde{\psi }}_{nb}^{n}$	${}^{\circ }$	[0.1;−0.1;−0.7]	[0.1;−0.1;−0.7]	[0.1;−0.1;−0.9]	[0.1;−0.1;−0.9]
50%/95% yaw	${}^{\circ }$	0.2/1.1	0.2/0.8	0.3/0.8	0.1/0.4
error quantile					
RMS of ${\tilde{v}}_{en}^{b}$	$\frac{\mathrm{cm}}{s}$	[6;−10;−5]	[5;−2;−4]	[6;−13;−5]	[7;−4;−6]
50%/95% hor.	$\frac{\mathrm{cm}}{s}$	6/23	3/10	8/29	5/16
quantile of ${\tilde{v}}_{en}^{b}$					
RMS of ${\tilde{v}}_{en}^{n}$	$\frac{\mathrm{cm}}{s}$	[9;−7;−4]	[6;−5;−4]	[4;−6;−4]	[5;−7;−5]

**Table 8:** Accuracy Metrics for Position Error, with and Without Odometry Aiding. GPS L1 Algorithm (First Line of Each Cell), Multi-Frequency GPS Algorithm (Second Line of Each Cell) and Multi-Frequency/Multi-Constellation Algorithm (Third Line of Each Cell). ENU Coordinate Frame, all Values in m.

Quantity	Data Set 1,	Data Set 1,	Data Set 2,	Data Set 2,
	Odometry Off	Odometry On	Odometry Off	Odometry On
Arithmetic mean of ${\tilde{p}}_{en}^{n}$	[−0.7;−0.5;−0.9]	[−0.8;−1.2;−0.2]	[−0.3;−0.4;−1.8]	[−0.4;−0.6;−2.1]
	[−0.6;−0.1;−1.2]	[−0.6;−0.6;−0.7]	[−0.2;−0.6;−0.3]	[−0.2;−0.5;−0.4]
	[−0.5;−0.2;−0.9]	[−0.4;−0.6;−0.6]	[−0.1;−0.4;−0.5]	[−0.1;−0.3;−0.6]
RMS of ${\tilde{p}}_{en}^{n}$	[−2.2;−2.8;−3.8]	[−1.8;−3.8;−3.6]	[−0.9;−1.5;−2.3]	[−1.3;−1.4;−2.4]
	[−2.1;−2.5;−4.1]	[−1.4;−2.8;−3.6]	[−0.9;−1.4;−1.3]	[−1.2;−1.2;−1.6]
	[−1.7;−2.1;−3.0]	[−1.2;−2.6;−2.7]	[−0.9;−1.3;−1.2]	[−1.0;−1.0;−1.5]
50%/95%	1.2/6.5	1.4/6.5	1.3/2.7	1.4/3.4
horizontal	1.3/8.2	1.3/4.4	1.0/2.7	1.2/3.0
quantile of ${\tilde{p}}_{en}^{n}$	1.0/6.2	1.1/4.2	0.9/2.5	1.0/2.5

**Table 9:** Quality Number Description and Associated 3-D Accuracy for Novatel’s Waypoint-Inertial Explorer [21].

Inertial Explorer Quality Number	Description	3-D Accuracy of Ref. Solution (in m)
1	Fixed integer	0.00–0.15
2	Converged float or noisy fixed integer	0.05–0.40
3	Converging float	0.20–1.00
4	Converging float	0.50–2.00
5	DGNSS	1.00–5.00
6	DGNSS	2.00–10.00

**Table 10:** RMS Position Error Depending on Reference Solution Quality. Multi-Frequency/Multi-Constellation Algorithm with Odometry Aiding, ENU Coordinate Frame.

Inertial Explorer Quality Number	Perc. of Epochs, Data Set 1 (in %)	RMS of ${\tilde{p}}_{en}^{n}$, Data Set 1 (in m)	Perc. of Epochs, Data Set 2 (in %)	RMS of of ${\tilde{p}}_{en}^{n}$, Data Set 2 (in m)
1	36	[1.0;−0.9;−2.4]	50	[0.7;−0.9;−1.3]
2	24	[1.0;−1.1;−2.5]	20	[0.8;−0.9;−1.4]
3	23	[0.9;−2.1;−2.4]	22	[1.0;−1.1;−1.6]
4	11	[1.1;−4.5;−3.5]	5	[1.9;−1.5;−2.4]
5	5	[3.0;−6.8;−4.6]	2	[3.0;−1.9;−2.6]
6	1	[1.7;−6.7;−3.6]	1	[1.2;−2.2;−3.7]
1–6	100	[1.2;−2.6;−2.7]	100	[1.0;−1.0;−1.4]

## Abbreviations

The following abbreviations are used in this manuscript:

BCU	brake control unit
BGD	Broadcast Group Delay
CAN	Controller Area Network
CCDIS	Crustal Dynamics Data Information System
CDF	cumulative distribution function
CDMA	code division multiple access
CNAV	civil navigation message
DCB	differential code bias
DOY	day of year
EKF	Extended Kalman Filter
ENU	east-north-up
ESC	electronic stability control
FLU	front-left-up
F/NAV	freely-accessible navigation message
GGTO	GPS to Galileo Time Offset
GNSS	global navigation satellite system
GPGA	GPS to Galileo time system correction
GPS	Global Positioning System
IF	ionosphere-free combination
IGS	International GNSS Service
IMU	inertial measurement unit
IONEX	Ionosphere Map Exchange Format
ISB	inter-system bias
ISC	inter-signal correction term
I/NAV	integrity navigation message
LNAV	legacy navigation message
MEMS	microelectromechanical systems
PDOP	position dilution of precision
PR	pseudo-range
rcv.	receiver
RINEX	Receiver Independent Exchange Format
RMS	root mean square
RTK	real-time kinematic positioning
SISRE	signal in space ranging error
std.	standard deviation
SV	space vehicle
TEC	total electron content
UEE	user equipment error
UERE	user equivalent range error
